# Differential Effects of Melatonin on Nitrogen Metabolism and Growth in *Capsicum chinense* Jacq.

**DOI:** 10.3390/plants15111713

**Published:** 2026-06-01

**Authors:** Fabiola León-García, Federico García-Laynes, Ruth Márquez-López, Fátima Medina-Lara, Camilo Escalante-Magaña, Adrián Toledo-Castiñeira, Ángel Córdova-Alvarado, Ileana Echevarria-Machado, Manuel Martinez-Estevez

**Affiliations:** Unidad de Biología Integrativa, Centro de Investigación Científica de Yucatán, Calle 43 #130 x 32 and 34, Mérida 97205, Yucatán, Mexico; ubi_fleon@cicy.mx (F.L.-G.); ubi_flaines@cicy.mx (F.G.-L.); ruth.marquez@cicy.mx (R.M.-L.); med72lmf@cicy.mx (F.M.-L.); ubi_cescalante@cicy.mx (C.E.-M.); adrian.toledo@estudiantes.cicy.mx (A.T.-C.); angel.cordova@estudiantes.cicy.mx (Á.C.-A.)

**Keywords:** melatonin, priming, nitrogen metabolism, *Capsicum chinense*

## Abstract

Melatonin has been recognized as a regulator of plant growth and stress responses; however, its role in nitrogen metabolism, particularly in *Capsicum chinense* Jacq., remains poorly understood. In this study, we evaluated the effect of melatonin priming alone (chemopriming, CP) or in combination with substrate reinforcement (soil drench, SD) (CP + SD), at different doses, on physiological, biochemical, and molecular parameters in habanero pepper plants growing in non-stressed conditions. Growth traits, nitrogen-related metabolites, total carbohydrates, the expression of key genes involved in nitrogen metabolism, and glutamate dehydrogenase (GDH) activity were analyzed. The results showed that priming increased biomass mainly at 25 and 50 µM, whereas the combined treatment promoted improvements from early doses (5 µM). Additionally, the evaluated genes exhibited tissue-specific expression patterns. Most genes were downregulated in leaves, whereas proline and total carbohydrate levels correlated with fresh and dry weight. Our results provide new insights into the regulation of nitrogen metabolism according to the application method and dosage, establishing an application strategy to optimize nitrogen metabolism and growth in habanero peppers. The mechanism of melatonin in regulating the balance of C and N metabolites and its impact on the response of chili plants to growth and environmental signals should be addressed later.

## 1. Introduction

Nitrogen (N) is one of the most important mineral nutrients for plant growth and productivity in agricultural systems [1]. This element is an essential component of amino acids, proteins, nucleic acids, chlorophyll, and numerous metabolites involved in fundamental processes of plant development and crop yield [2,3]. Despite its importance, N availability in the soil is often a limiting factor in agricultural production [4], which has led to the intensive use of nitrogen fertilizers. However, the excessive application of these fertilizers has generated significant agronomic and environmental problems, including soil salinization, nutritional imbalances, reduced nitrogen use efficiency, and aquifer contamination [5,6,7].

N can be supplied to plants in inorganic form such as nitrate (NO_3_^−^) and ammonium (NH_4_^+^) or organic form (amino acids and urea). However, nitrate is the main source of N absorbed by roots and utilized by most cultivated species [8,9]. N metabolism in plants comprises a series of highly coordinated physiological and biochemical processes, including the absorption, reduction, assimilation, and redistribution of nitrate. Once inside the plant, nitrate is reduced by nitrate reductase (NR) and subsequently to ammonium by nitrite reductase (NiR) [3]. The ammonium generated is incorporated into organic compounds through the glutamine synthetase/glutamate synthase (GS/GOGAT) pathway or by glutamate dehydrogenase (GDH) activity [3,10,11]. These metabolic processes regulate amino acid and protein synthesis and are closely related to plant growth and nitrogen use efficiency.

Various endogenous signals, including plant hormones, redox signals, and transcriptional and post-translational regulatory mechanisms, coordinate the dynamic adjustment of N uptake and assimilation [4]. In recent years, there has been a growing interest in identifying signaling molecules capable of coordinating N metabolism with other key physiological processes. Among these molecules, melatonin (N-acetyl-5-methoxytryptamine), initially identified as a hormone in animals [12], is known as a multifunctional regulator in plants [13,14]. It has been detected in numerous plant tissues, associated with multiple physiological processes, including the regulation of growth, root system architecture, photosynthesis, stress tolerance, among others [15,16,17,18,19].

Melatonin has been widely recognized for its role in enhancing plant growth from early developmental stages. Several studies have shown that pretreatment with low concentrations of melatonin improves seed germination. This effect has been attributed to increased activity of antioxidant enzymes and hormonal modulation, particularly by decreasing abscisic acid levels [20]. Similarly, applying low concentrations of melatonin to *Stevia rebaudiana* Bertoni seeds promotes plant morphological development, increasing fresh weight and the number of leaves [21].

In addition to its effects during germination, melatonin also promotes root system development, which is key for water and nutrient uptake. Concentrations of 10–30 µM of melatonin have been reported to increase root system vigor, root hair growth, and lateral root formation in tomato [22]. Furthermore, melatonin induces the formation of root primordia, modifying the length and number of adventitious and lateral roots [23].

Recent evidence suggests that melatonin plays a significant role in regulating N metabolism. Exogenous melatonin administration has been reported in several species to improve N uptake and metabolism, increase the expression and activity of key N metabolism enzymes [24,25,26], and enhance nitrogen use efficiency [27]. These effects appear to be associated with melatonin’s ability to modulate cellular redox status, regulate the expression of genes related to N metabolism, and coordinate the interaction between carbon and N metabolic pathways [28,29,30].

The family includes approximately 100 genera and more than 2500 species widely distributed in tropical and temperate regions of the world [31]. Within this family, the genus *Capsicum* comprises around 35 species [32] of great agricultural, economic, and cultural importance globally. Among them, tomato (*Solanum lycopersicum*), potato (*Solanum tuberosum*), and eggplant (*Solanum melongena*), along with *Capsicum* species [33], constitute relevant models for the study of physiological and metabolic processes in plants. In particular, *Capsicum chinense* Jacq., known as habanero pepper, is among the top five *Capsicum* species due to its high commercial value and its relevance in gastronomy [32]. This species is valued for its high capsaicinoid content, compounds responsible for its pungency, as well as for its richness in bioactive metabolites with nutritional and pharmacological potential [34]. Fruit yield and quality are influenced by soil nutrient availability, especially N, which impacts both the production and accumulation of secondary metabolites and gene expression [35,36]. Likewise, the growing conditions of the habanero pepper, such as soil type, influence plant growth and development [37].

In this context, the use of growth regulators such as melatonin represents a potential strategy to improve the physiological and metabolic performance of *Capsicum* species under different environmental conditions. In *Capsicum annuum*, melatonin improves stress tolerance by activating key enzymes in N metabolism, thus promoting N assimilation [38]. Furthermore, in this species, melatonin has been shown to reduce oxidative damage and regulate redox status, contributing to a better response to abiotic stress conditions [39]. Additionally, melatonin has also been reported to promote root growth and improve stress tolerance in seedlings by activating the antioxidant system and regulating osmotic adjustment [40].

Despite growing interest in melatonin as a plant growth regulator, there is still limited information on its role in regulating nitrogen metabolism in agriculturally important species. Most studies on the effect of melatonin on N metabolism are conducted under abiotic stress conditions. In these studies, the use of different application methods and doses of melatonin has led to discrepant results, even within the same species [41]. Although recent studies have suggested, through in silico approaches, the possible existence of melatonin receptors in habanero peppers [42], the physiological, biochemical, and molecular mechanisms by which melatonin modulates N assimilation remain unknown, especially in response to different application concentrations.

In this study, we took advantage of the lack of knowledge regarding the impact of different application methods and dosages of melatonin on the regulation of nitrogen metabolism in plants growing under non-stressful conditions. We hypothesized that under these growth conditions, the application method would differentially modify nitrogen metabolism through specialized mechanisms, leading to specific impacts on habanero pepper growth for each treatment. The results obtained in this research suggest that melatonin application can be used as a strategy to optimize nitrogen metabolism and the growth of habanero peppers.

## 2. Results

### 2.1. Melatonin Effect on Habanero Pepper Growth

To determine the effect of melatonin on the growth of habanero pepper seedlings, we used two approaches. In the first, the seeds were soaked in increasing concentrations of melatonin for 72 h (chemopriming, CP), while in the second, two additional melatonin applications were administered to the plant substrate (soil drench, SD), maintaining the same dose at which these seeds had previously been soaked (CP + SD). Priming with melatonin did not affect either the seed germination rate or the total germination percentage.

**Figure 1:**
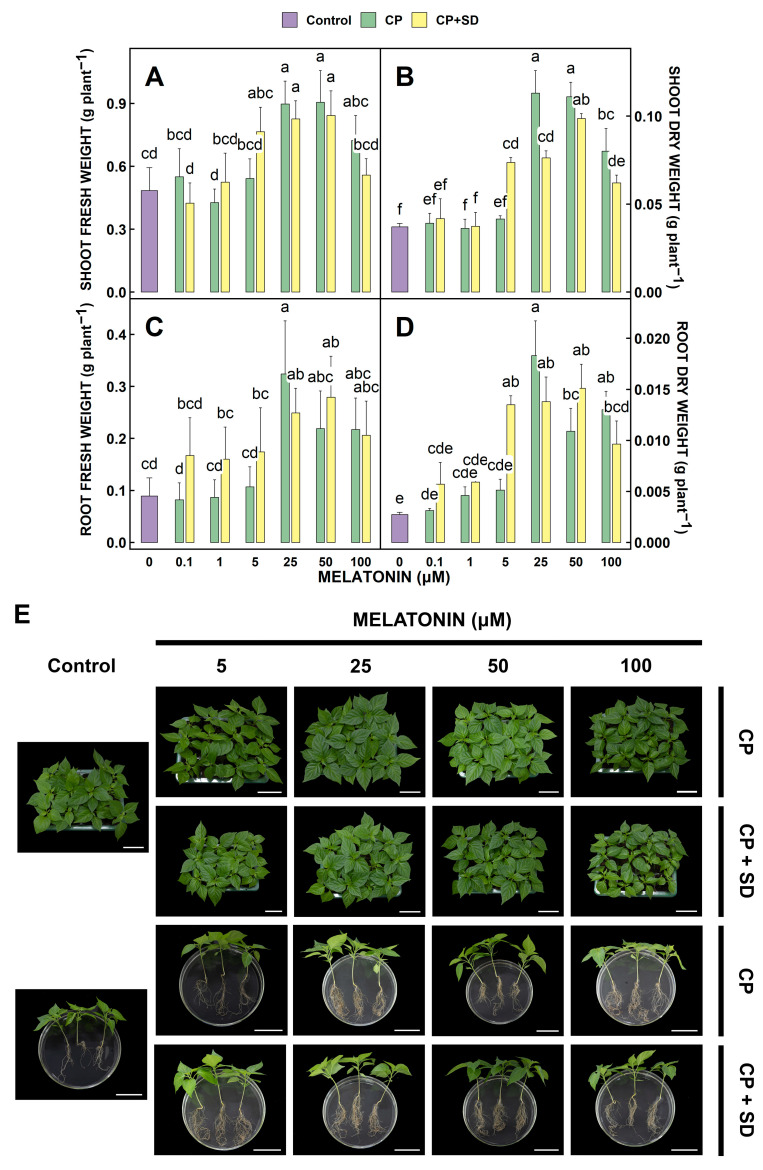
Growth response of *C. chinense* plants to melatonin treatment. Fresh (**A**) and dry (**B**) shoot weight, fresh (**C**) and dry (**D**) root weight, and morphological changes in the plants (**E**). The photographs at the top in (**E**) show the plants growing into 12-well trays, while the lower ones show three representative plants from each treatment, once they were collected from the pots, as described in materials and methods. Data represent mean values ± standard deviation (SD) (*n* = 9). Different letters indicate significant differences among treatments according to Tukey’s (*p* ≤ 0.05).

The effect of melatonin on the growth of habanero pepper plants was dose- and application-dependent. The CP treatment resulted in a significant increase in the fresh and dry weight of the plant shoots, starting at a dose of 25 µM melatonin, which was maintained up to doses of 50 and 100 µM, respectively (Figure 1A,B). The 25 µM dose stimulated root growth, both in terms of fresh and dry weight, when the CP treatment was applied, and this effect continued to be significant on root dry weight at higher doses (Figure 1C,D).

Interestingly, the CP + SD treatment did not produce significantly greater stimulation than seed priming with melatonin alone, except for the 5 µM, where higher shoot and root dry weights were observed (Figure 1B,D).

Figure 1E shows the foliar and root development of the plants treated with melatonin via CP, where a maximum stimulatory effect is evident, especially at doses of 25 and 50 µM. This stimulatory effect was not surpassed by the CP + SD treatment.

### 2.2. Melatonin Effect on Nitrate, Ammonium, Total Amino Acid, and Total Protein Levels

The effect of melatonin on nitrogen metabolite content was dependent on the form of application, the concentration, and the plant organ.

**Figure 2:**
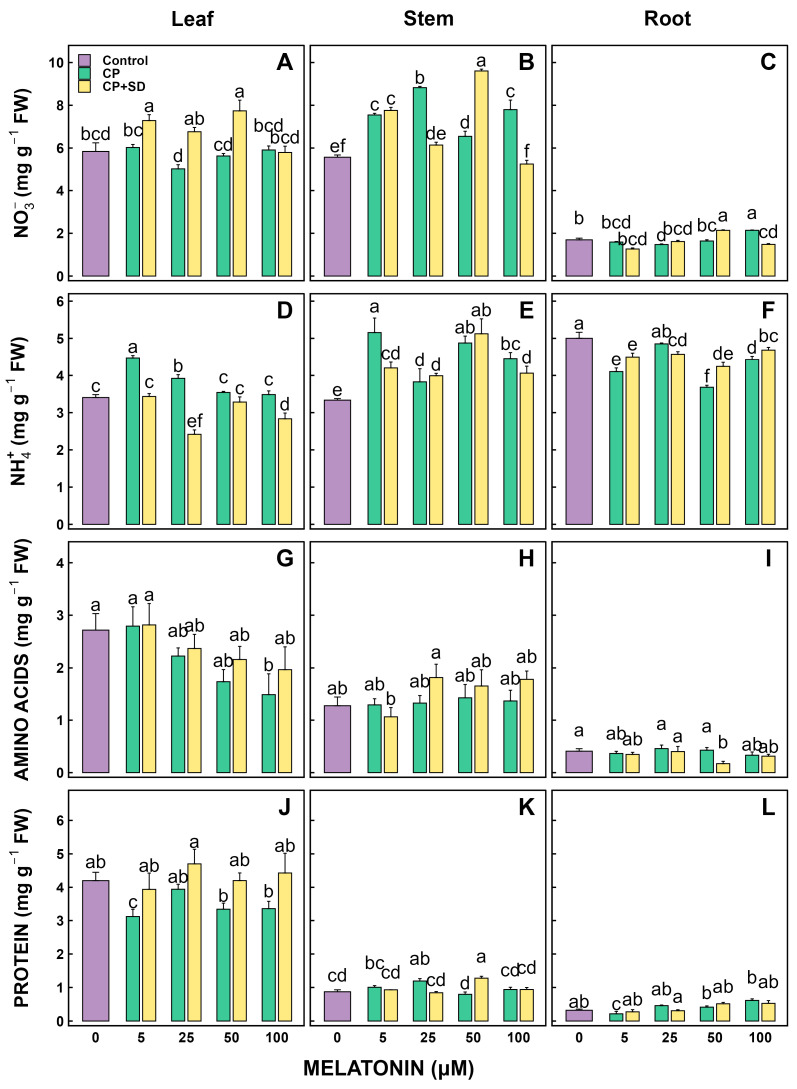
Effect of melatonin treatments on nitrogen-related metabolites in *C. chinense.* Nitrate content (**A**–**C**), ammonium content (**D**–**F**), amino acid content (**G**–**I**), and protein content (**J**–**L**) were determined in leaves (**A**,**D**,**G**,**J**), stems (**B**,**E**,**H**,**K**), and roots (**C**,**F**,**I**,**L**). Data represent mean values ± SD (*n* = 9). Different letters indicate significant differences among treatments according to Tukey’s (*p* ≤ 0.05). FW: Fresh Weight.

In leaves, the highest nitrate content was obtained with the CP + SD treatment at doses between 5 and 50 µM. Only the CP application did not significantly affect these values (Figure 2A). In contrast, nitrate content in the stem was significantly higher with the CP treatment, reaching its maximum at a dose of 25 µM. These values were exceeded when melatonin was applied in addition to seed priming at a dose of 50 µM (Figure 2B). Nitrate content in the root was lower than that found in the aerial parts. The effect of both melatonin application methods on endogenous root nitrate content was dose-dependent. Lower doses reduced levels (25 and 5 µM for CP and CP + SD, respectively) and higher doses stimulated them (100 and 50 µM for CP and CP + SD, respectively) (Figure 2C).

Ammonium content in leaves increased significantly with the CP treatment at doses of 5 and 25 µM, while it decreased with the CP + SD treatment at 25 and 50 µM (Figure 2D). In the stem, all treatments increased ammonium content (Figure 2E), while in the root it decreased (Figure 2F).

Regarding amino acid content in the leaves, a decrease in these values was observed with increasing melatonin concentration in the CP treatment, reaching values significantly lower than 100 µM. Interestingly, the CP + SD treatment did not cause significant changes in this parameter (Figure 2G). In the stem, total amino acid content did not vary significantly with the treatments used (Figure 2H), while in the root, a significant decrease was only observed in the CP treatment at 50 µM melatonin (Figure 2I).

Significantly, endogenous total protein content in leaves decreased in the CP treatment at doses of 5, 50, and 100 µM, while it was unaffected in the CP + SD treatment (Figure 2J). In the stem, these contents increased in both treatments, but at different doses: 25 µM in CP and 100 µM in CP + SD (Figure 2K). In the root, total protein only decreased significantly in the CP treatment at 5 µM melatonin (Figure 2L).

When the sum of the different forms of nitrogen (nitrate + ammonium + amino acids + protein) was performed by plant organ, the greatest changes with respect to the control were observed in the stem, with the highest values reaching the CP + SD treatment at a dose of 50 µM (Figure S1).

### 2.3. Principal Component Analysis (PCA) of Nitrogen Related Metabolites in Response to Melatonin

**Figure 3:**
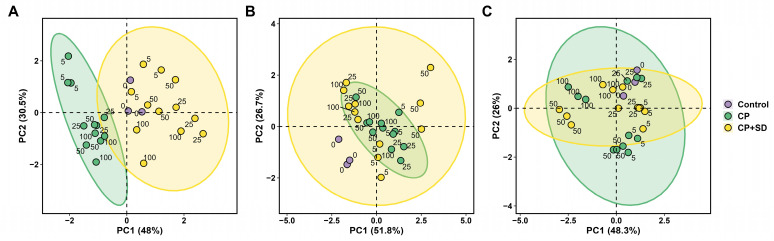
Principal component analysis (PCA) based on nitrate, ammonium, amino acids, and protein contents measured in different plant organs under the evaluated treatments. Panels (**A**), (**B**), and (**C**) represent independent PCA analyses for leaf, stem, and root, respectively. Colored ellipses indicate the 95% confidence interval for each treatment group; tighter ellipses indicate greater similarity among samples within a treatment, whereas broader ellipses reflect higher variability in the multivariate response. Numbers associated with each point correspond to the applied melatonin concentrations.

The principal component analysis (PCA) shown in Figure 3 was performed using variables associated with N metabolism, including nitrate, ammonium, amino acids, and protein contents in leaves, stems, and roots under different melatonin treatments. Overall, the first two principal components explained a high proportion of the total variance.

In leaves (Figure 3A), PC1 explained 48% of the total variance, whereas PC2 accounted for 30.5%. A clear separation was observed between the CP and CP + SD treatments. CP samples were grouped toward negative PC1 values, while CP + SD were distributed toward positive PC1 values, suggesting differential metabolic responses in N metabolism between treatments. The increased melatonin concentrations in CP + SD also promoted the formation of clusters within each treatment group, indicating a dose-dependent effect on the accumulation of nitrogen compounds.

In stems (Figure 3B), PC1 explained 51.8% of the total variance and PC2 explained 26.7%. Control samples tended to group toward negative PC1 values and CP- and CP + SD-treated plants toward positive PC1 values, suggesting that both treatments induced differential metabolic responses. However, unlike leaves, the stem showed a greater overlap among CP and CP + SD treatments, indicating a lower degree of metabolic differentiation.

In roots (Figure 3C), PC1 and PC2 explained 48.3% and 26% of the total variance, respectively. In contrast to leaves, root samples did not show a clear separation by treatment, with substantial overlap among the control, CP, and CP + SD groups. This distribution pattern suggests that the evaluated treatments induced minor changes in root nitrogen metabolic profile. On the other hand, melatonin concentrations did not generate a consistent clustering pattern, indicating that root metabolic responses remained homogeneous. Overall, the PCA suggests that leaves were more sensitive to treatment-induced modifications in nitrogen metabolism, while stems and roots maintained more stable metabolic profiles.

### 2.4. Phylogenetic Analyses of Nitrogen Assimilation Enzymes in C. chinense

**Figure 4:**
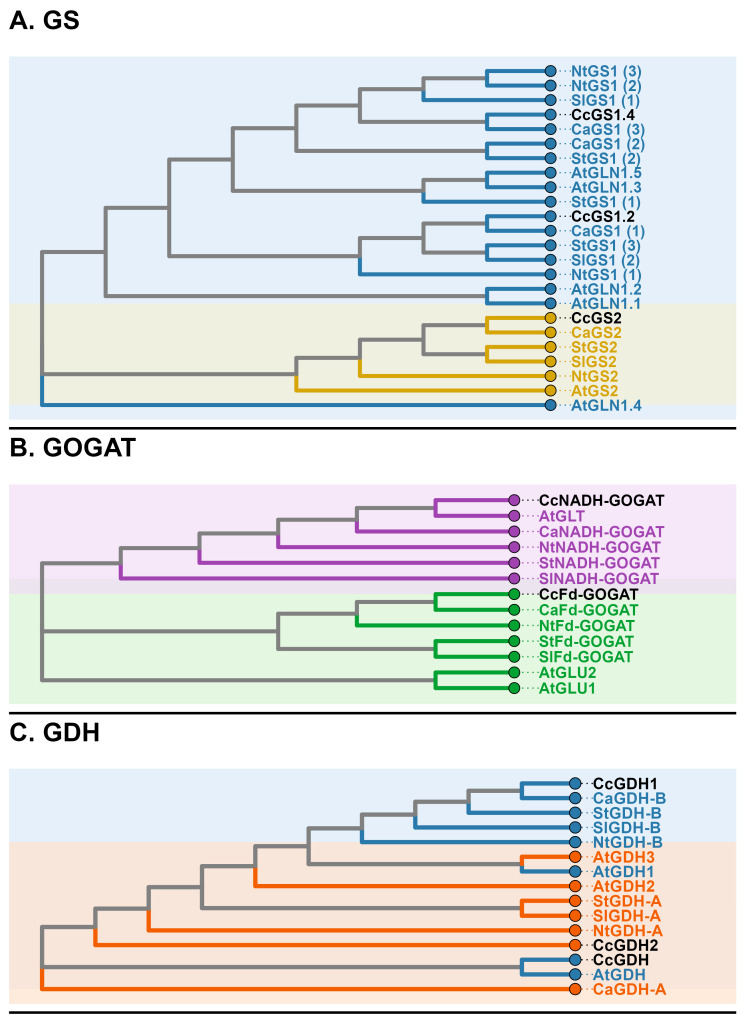
Phylogenetic analysis of nitrogen metabolism-related enzymes in *C. chinense.* Phylogenetic trees were constructed based on amino acid sequence alignments of (**A**) GS, (**B**) GOGAT, and (**C**) GDH from *C. chinense* and their homologs from representative Solanaceae species (*S. lycopersicum*, Sl; *S. tuberosum*, St; *C. annuum*, Ca; and *Nicotiana tabacum*, Nt), as well as *Arabidopsis thaliana*, At. The analysis was performed using MEGA12. Trees were constructed using Neighbor-Joining method with the p-distance model. Gaps and missing data were treated using pairwise deletion. Putative *C. chinense* proteins identified in this study are highlighted in black.

Phylogenetic analyses were conducted to characterize the GS, GOGAT, and GDH proteins families identified in *C. chinense*. In the GS phylogenetic tree (Figure 4A), three *C. chinense* sequences were distributed into two clearly defined clades corresponding to the major GS isoforms. Two sequences, CcGS1.2 and CcGS1.4, clustered within the cytosolic GS1 clade, whereas CcGS2 grouped within the plastidial GS2 clade, supporting the presence of both cytosolic and plastidial isoforms in habanero pepper.

The GOGAT phylogeny (Figure 4B) revealed the presence of two distinct sequences that segregated into the canonical functional classes of GOGAT. CcFD-GOGAT clustered within the ferredoxin-dependent GOGAT (Fd-GOGAT) clade and showed a close phylogenetic relationship with *C. annuum* CaFd-GOGAT (Figure 4B). CcNADH-GOGAT grouped within the NADH-GOGAT clade together with AtGLT (*A. thaliana* NADH-GOGAT).

Similarly, phylogenetic analysis of GDH proteins (Figure 4C) identified three *C. chinense* sequences distributed into distinct clades within the GDH family. CcGDH clustered closely with *A. thaliana* GDH (AtGDH), whereas CcGDH1 grouped with *C. annuum* GDH-B (CaGDH-B). In contrast, CcGDH2 formed a separate clade closely related to CcGDH. Sequence nomenclature was assigned based on the closest phylogenetic relationships, and in cases where phylogenetic resolution was insufficient, sequence identity percentages (Table 1) were additionally considered to support protein classification.

### 2.5. Expression of Genes Associated with Nitrogen Metabolism in Response to Melatonin Treatments

To further investigate the effect of melatonin on nitrogen metabolism, semiquantitative reverse transcription polymerase chain reaction (RT-PCR) analyses were performed as an initial screening, followed by a quantitative reverse transcription (RT-qPCR). For RT-PCR, root and leaf samples from plants treated with 25 and 50 µM melatonin were used, while for the quantitative analysis, only the treatments with a dose of 25 µM were selected.

**Figure 5:**
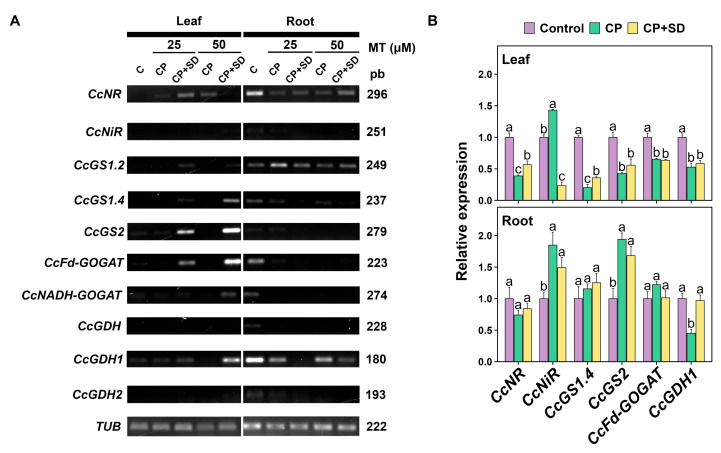
Transcription patterns of nitrogen metabolism-related genes in *C. chinense* plants treated with melatonin, as revealed by endpoint RT-PCR (**A**) and RT-qPCR analysis (**B**). Relative expression levels were calculated using the 2^−ΔΔCT^ method. Data represent mean values ± SD. Different letters indicate significant differences among treatments for each gene according to Tukey’s (*p* ≤ 0.05). *NR*, nitrate reductase; *NiR*, nitrite reductase; *GS*, glutamine synthetase; *GOGAT*, glutamate synthase; *GDH*, glutamate dehydrogenase.

These analyses revealed differential modulation of gene expression depending on the organ and application regimen (Figure 5A). In leaves, seven of the ten genes evaluated (*CcNR*, *CcGS1.4*, *CcGS2*, *CcGS1.2*, *CcGDH1*, *CcFd-GOGAT*, and *CcNADH-GOGAT*) showed greater intensity under CP + SD (Figure 5A). In roots, most genes exhibited more conserved patterns; *CcNiR*, *CcGDH*, and *CcNADH-GOGAT* showed no detectable expression (Figure 5A). *CcNR* showed a concentration- and organ-dependent response: in leaves, the highest expression was observed in plants treated with 25 µM, while at 50 µM the pattern was reversed; in roots, the most evident increase occurred in plants subjected to 50 µM (Figure 5A).

Members of the GS family showed a general tendency to increase their expression in leaves under CP + SD, especially at 50 µM. In roots, *CcGS1.4* and *CcGS2* maintained comparable levels between the control and the 25 µM treatment under CP, while *CcGS1.2* showed a more uniform pattern (Figure 5A). In the GDH family, *CcGDH* and *CcGDH2* were detectable only in roots of the control, while *CcGDH1* showed a differential response, with higher expression in roots under the 50 µM CP treatment and an opposite pattern in leaves at 25 µM (Figure 5A).

*CcFd-GOGAT* and *CcNADH-GOGAT*, on the other hand, showed organ-dependent regulation, with higher expression in leaves under certain enhanced treatments, while in roots their expression was limited or restricted to the control. The results suggest that the transcriptional response induced by melatonin was modulated by both the applied concentration and the organ and application schedule.

Based on these results, we selected the dose of 25 µM and the *CcNR*, *CcNiR*, *CcGS1.4*, *CcGS2*, *CcFd-GOGAT*, and *CcGDH1* genes for real-time PCR studies. The expression of *CcNR* showed a tissue-dependent response. In leaves, the transcription levels of this gene decreased 2.6 and 1.8 times in the CP and CP + SD melatonin treatments, respectively, compared to the control plants. In contrast, no significant differences were observed between treatments in roots (Figure 5B).

For *CcNiR*, a differential pattern was observed between tissues. In leaves, melatonin priming induced a 1.4-fold increase in the transcription levels of this gene compared to the control, while the CP + SD treatment reduced its expression 4.3-fold (Figure 5B). In roots, both treatments showed expression levels 1.8 and 1.5 times higher than the control (Figure 5B). Regarding the genes involved in ammonium assimilation, the expression of *CcGS1.4* decreased 4.9 and 2.8-fold in leaves in the CP and CP + SD treatments, respectively, while no significant differences between treatments were detected in roots (Figure 5B). Conversely, *CcGS2* showed an opposite pattern between tissues. In leaves, the expression of this gene was 2.4 and 1.8 times lower in plants treated with melatonin compared to the control, while in roots, transcription levels increased 1.9 and 1.7 times in the CP and CP + SD treatments, respectively (Figure 5B).

Similarly, *CcFd-GOGAT* expression decreased 1.5 and 1.6 times in leaves in response to the CP and CP + SD treatments, respectively, compared to control plants, while no significant differences were observed between treatments in roots (Figure 5B). *CcGDH1* expression also showed a tendency to decrease in leaves under the CP and CP + SD treatments, by 1.9 and 1.7 times, respectively, while in roots, a 2.2-fold decrease in expression was observed in CP plants (Figure 5B).

Taken together, these results indicate that melatonin treatments modulate the expression of genes associated with nitrogen metabolism in a tissue-dependent manner, characterized by an overall decrease in expression in leaves and differential changes in roots.

### 2.6. Multivariate Analysis of Nitrogen Metabolism Gene Expression Under Melatonin Treatments

**Figure 6:**
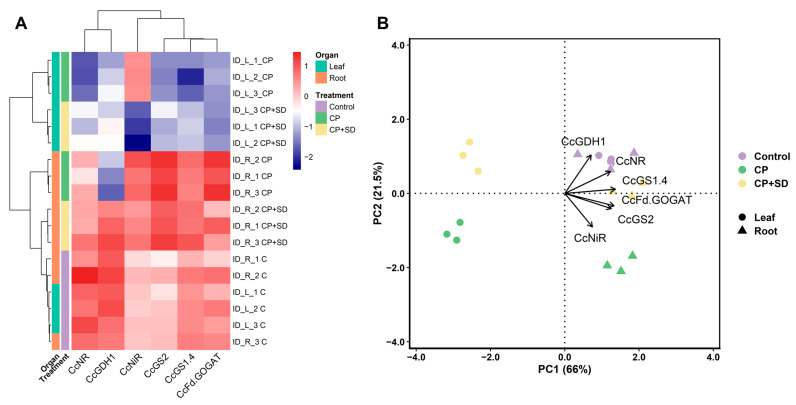
Multivariate analysis of the relative expression of nitrogen metabolism-related genes in leaves and roots of plants subjected to different treatments of melatonin. (**A**) Heatmap with hierarchical clustering based on the expression levels of *CcNR*, *CcGDH1*, *CcNiR*, *CcGS2*, *CcGS1.4*, and *CcFd-GOGAT*. Red tones indicate higher relative expression and blue tones indicate lower expression. Side annotations indicate the analyzed organ (leaf and root) and treatment (Control, CP, and CP + SD). (**B**) Principal component analysis (PCA) performed using gene expression data. Symbols represent organs (circles: leaves; triangles: roots), while colors correspond to treatments. Arrows indicate the contribution of each gene in sample separation.

The multivariate analyses revealed clear differences in the expression patterns of nitrogen metabolism genes between organs and treatments (Figure 6). In the heatmap analysis (Figure 6A), samples were grouped according to organ type, showing a marked separation between leaves and roots. Root samples generally exhibited higher expression levels of *CcNiR*, *CcGS2*, *CcGS1.4*, and *CcFd-GOGAT*, particularly under CP and CP + SD treatments. In contrast, leaf samples showed lower expression levels for most genes, especially under CP + SD conditions, where strong downregulation was observed for *CcNiR*. Control samples from both organs clustered separately from treated CP and CP + SD treatments, suggesting that the treatments induced transcriptional changes in genes associated with nitrogen metabolism.

The PCA supported the clustering observed in the heatmap (Figure 6B). PC1 and PC2 explained 66% and 21.5% of the total variance, respectively. Samples were separated mainly according to treatment and organ. Leaves were grouped on the negative side of PC1, whereas roots were displaced toward positive PC1 values, indicating organ associated transcriptional responses. Samples also showed differentiation among treatments, with CP samples tending to cluster at negative PC2 values and CP + SD at positive PC2 values. The vectors indicated that *CcGS1.4*, *CcFd-GOGAT*, and *CcGS2* contributed strongly to sample separation along PC1, while *CcGDH1* and *CcNiR* were more associated with variation along PC2.

### 2.7. Melatonin Enhances *GDH* Activity in C. chinense

**Figure 7:**
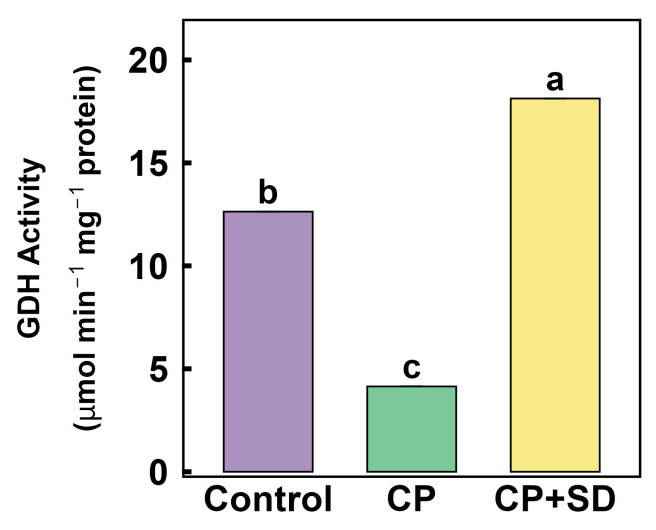
Effect of melatonin application on GDH activity in leaf of *C. chinense* plants. Data represent mean values ± SD. Different letters indicate significant differences among treatments for each gene according to Tukey’s (*p* ≤ 0.05).

GDH activity showed notable differences among treatments (Figure 7). Plants subjected to CP treatment showed a marked reduction in GDH activity compared with the control plants. In contrast, the CP + SD treatment induced the highest GDH activity values, exceeding both the control and CP treatments.

### 2.8. Effect of Melatonin on Carbohydrate and Proline Accumulation in Habanero Pepper Plants

**Figure 8:**
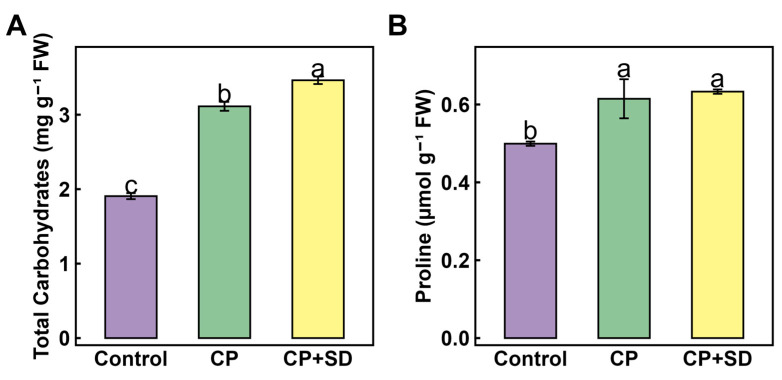
Effect of melatonin treatments on carbohydrate and proline content in *C. chinense* plants. (**A**) Total carbohydrates and (**B**) Proline content. Data represent mean values ± SD. Different letters indicate significant differences among treatments for each gene according to Tukey’s (*p* ≤ 0.05).

Treatment with CP and CP + SD significantly affected the accumulation of carbohydrates and proline in habanero pepper plants (Figure 8). Total carbohydrate content increased markedly in response to both treatments compared with the control plants. The highest carbohydrate content was observed with the treatment CP + SD (Figure 8A).

Proline accumulation was also stimulated by CP and CP + SD treatments (Figure 8B). Treated plants exhibited higher proline levels than control plants.

### 2.9. Relationship Between Nitrogenous Metabolites, Gene Expression, *GDH* Activity, Proline, Carbohydrates, and Growth Parameters in Melatonin Treatments

Pearson correlation analysis revealed strong associations among physiological, biochemical, and molecular variables related to nitrogen metabolism in *C. chinense* plants (Figure S2). Hierarchical clustering grouped the evaluated variables into three main clusters according to their correlation patterns.

A first cluster included amino acids and the expression levels of nitrogen metabolism-related genes (*CcFd-GOGAT*, *CcGS1.4*, *CcGDH1*, *CcNR*, and *CcGS2*), which showed strong positive correlations with each other (r = 0.90–0.98). Amino acid content was also positively correlated with these genes (r = 0.62–0.74), suggesting coordinated regulation of nitrogen assimilation pathways.

A second cluster comprised nitrate content, GDH enzymatic activity, and protein accumulation. Nitrate content was strongly positively correlated with GDH activity (r = 0.92) and protein content (r = 0.70). In contrast, ammonium levels and *CcNiR* expression were strongly negatively correlated with nitrate and GDH activity (r = −0.90 to −0.95), but positively associated with each other (r = 0.99), indicating an inverse relationship between nitrate assimilation and ammonium accumulation.

A third cluster grouped proline, carbohydrates, shoot dry weight, and shoot fresh weight, which displayed strong positive correlations among themselves (r = 0.67–0.90). These variables were negatively correlated with amino acids and nitrogen metabolism-related genes (r = −0.62 to −0.96), suggesting that metabolic adjustments associated with osmoprotection and biomass accumulation may occur independently from transcriptional activation of nitrogen assimilation genes.

**Figure 9:**
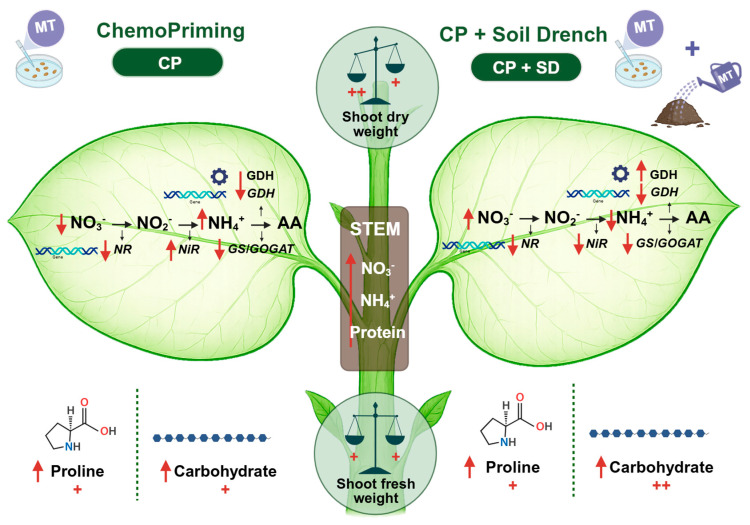
Proposed model summarizing the effects of melatonin application through CP and CP + SD on nitrogen metabolism and physiological responses in habanero pepper plants. Created with BioRender (https://BioRender.com).

In summary, these results are associated with low nitrate and GDH activity levels, as well as high ammonium and *CcNiR* transcript levels in the leaves of plants in the CP treatment versus high nitrate and GDH activity levels, and low ammonium and *CcNiR* transcript levels in those of the CP + SD treatment (Figure 9).

## 3. Discussion

### 3.1. Interrelationship Between Forms of Melatonin Application, Growth, Nitrogen Metabolism and Carbohydrates in Habanero Peppers Growing Under Non-Stressful Conditions

The central role of melatonin in regulating N metabolism under different abiotic stresses has been demonstrated in numerous plant species such as tobacco [25], cucumber [43], pepper [38], and wheat [1], among others. Although previous studies have made valuable contributions to the understanding of melatonin’s role in plant growth, critical limitations remain in experimental designs (application methods, effective doses, etc.) and in mechanistic studies, especially those related to nitrogen metabolism. We directed this research toward topics that have been little explored to date: the interaction of different doses of melatonin with growth and nitrogen metabolism under non-stressful growing conditions. We investigated the effect of the application method on this interaction. The model system used was the habanero pepper, a solanaceous species not yet studied in this field, which grows in the Yucatan Peninsula in nitrogen-poor soils [44]. This type of study can provide information on how melatonin could optimize the growth of this species by regulating nitrogen metabolism.

Our results showed that both forms of melatonin application, via CP or CP + SD, increased the growth of habanero pepper, based on FW or dry weight (DW), with an effective dose of 25–50 µM. Notably, the CP treatment was better at increasing the dry weight of the plants. Similar growth-promoting effects of melatonin have been widely reported in several plant species. In common bean, melatonin priming stimulates both shoot and root development, increasing plant length and biomass even under magnesium deficiency [45]. Likewise, melatonin-pretreated tomato seeds exhibit enhanced growth performance, including increased shoot length, stem diameter, and overall biomass [46]. Seed priming with 50 µM melatonin in peanut enhances plant growth, even in the absence of stress conditions [47].

The combination of priming with subsequent applications (CP + SD) showed effects at lower concentrations (5 µM), at which only seed priming failed to show any effect. This combined effect has been little explored, but recent studies suggest that the combined application of 100 µM melatonin via priming and foliar spraying can help mitigate stress in *Brassica juncea* L. by increasing various growth parameters such as shoot length, fresh weight, and dry weight, among others [48].

The application of 150 µM melatonin to the nutrient solution of tomatoes grown hydroponically did not alter plant growth [41]. A similar behavior was observed when 100 µM melatonin was applied by foliar spray to *C. annuum* plants [38]. However, foliar spraying of 100 µM melatonin to tomato plants, also grown hydroponically, increased their fresh and dry weight [49].

The application method of melatonin had specific effects on nitrogen metabolism parameters in habanero peppers. While both application methods increased stem nitrogen content, specifically in the form of nitrate and ammonium, indicating greater translocation to the leaves, the leaf was the most sensitive organ to the modifications induced by the melatonin treatments on nitrogen metabolites. The CP application method resulted in a decrease in nitrate and an increase in ammonium in the leaves, while the opposite effect was observed in the CP + SD treatment. This result suggests that the application method of melatonin can induce fine-tuning of nitrogen metabolism in the leaf, optimizing its use for dry matter production.

Studies evaluating the effect of different forms of melatonin application on these nitrogen sources in different plant organs under non-stressful growth conditions are lacking. In alfalfa, melatonin application under high-nitrogen conditions decreased nitrate-N and ammonium-N concentrations compared to untreated plants [50]. Applying 50 µM of melatonin via hydroponic root drenching to tobacco decreased nitrate and ammonium levels under high nitrogen stress, by increasing the expression of the *NR*, *NiR*, *GS*, *GOGAT*, and *GDH* genes. This resulted in a recovery of growth under this stressful condition. These authors found that the melatonin-induced transcription factor NtbHLH96 was responsible for upregulating genes related to nitrogen metabolism but did not alter photosynthetic genes [25]. Similarly, foliar application of melatonin also reduces nitrate, ammonium, and free amino acid levels in sweet pepper plants subjected to water stress. This led to a decrease in oxidative stress, activation of nitrogen metabolism enzymes, and recovery of growth [38].

However, it is necessary to point out that the stimulatory effect of melatonin on gene expression, the activity of primary nitrogen metabolism enzymes, and the reduction of nitrate and ammonium that leads to increased growth is a phenomenon that occurs under stressful conditions, but in most cases does not repeat itself under non-stressful conditions [25,38,41].

We hypothesize that the lack of growth stimulation, as well as the absence of effect of melatonin on nitrate and ammonium content in other solanaceous plants such as tomato, tobacco and *C. annum* reported in previous studies, is due to the dose used and the method of application [25,38,41]. These studies used single doses of melatonin of between 100 and 150 µM which, while effective in alleviating the stress to which the plants were subjected, were unable to increase growth or modify the levels of these metabolites under non-stressful conditions. This result coincides with the results here, where the stimulatory effect on habanero pepper growth is reduced or disappears at doses greater than 50 µM. The application method must also be considered. Even using the same species, tomato, and exhibiting similar behavior under salt stress conditions, the results varied under non-stressful conditions when melatonin was applied via foliar spray versus mixed with the nutrient solution in the hydroponic medium [41,49].

Through the approach taken here, it is demonstrated that reinforcing the application of melatonin to the substrate, in addition to seed chemopriming, does not further modify the remobilization of inorganic nitrogen from the root to the leaves beyond that which occurs with chemopriming alone. However, both application methods do lead to significant differences in leaf nitrogen metabolism, the impact of which on plant responses to different signals should not be disregarded.

Interestingly, proline content increased to the same extent in both forms of melatonin application at a dose of 25 µM, while total carbohydrates also increased, but with the booster application, their values were higher. The synthesis of soluble compounds, such as proline and sugars, to maintain plant water status under stress conditions has been widely demonstrated [38,51]. Proline not only acts as an osmoprotectant but also neutralizes reactive oxygen species [52].

The mechanism by which melatonin increases proline levels is not well understood. However, Ref. [41] recently demonstrated that melatonin-induced proline accumulation in tomato plants growing under salt stress was reversed using a nitric oxide scavenger. These results confirmed the important regulatory role of melatonin-induced nitric oxide in regulating proline metabolism.

The availability of carbohydrates produced during photosynthesis is essential for plant growth [53]. The role of melatonin in regulating carbohydrate metabolism is well-established. Melatonin increases the transcriptional activity of several enzymes associated with photosynthesis, sucrose, starch, and other metabolic pathways [54]. Foliar application of melatonin to tomatoes, growing under non-stressful conditions, increased sucrose content, the expression of sucrose transporters (SUT1, 2, and 4), the large (rbcL) and small (rbcS) subunits of Rubisco, and other genes related to carbohydrate metabolism. Melatonin can also increase photosynthetic capacity in various crops under stress by protecting chlorophyll [25].

The melatonin-mediated accumulation of proline and carbohydrates in habanero pepper plants growing under non-stressful conditions may represent an additional mechanism for protecting the plant from future stress. Since these metabolites, unlike inorganic forms of nitrogen, showed a high correlation with growth values based on fresh and dry weight, future studies should focus on the mechanisms by which melatonin regulates their accumulation in habanero pepper leaves and their role under stress conditions.

### 3.2. Interrelationship Between Forms of Melatonin Application, Growth, Transcriptional and Enzymatic Responses in Habanero Peppers Growing Under Non-Stressful Conditions

NR and NiR collaborate to convert nitrate taken up by the plant to ammonium [55], while GDH, GS and GOGAT participate in the synthesis of amino acids from which nitrogenous macromolecular compounds are derived [56].

The effect of melatonin on transcript levels and enzyme activity has been studied in several species under stress. In general, melatonin application increases transcript levels and enzyme activity, regulating nitrate, ammonium, and amino acid levels, thereby alleviating stress from high nitrogen concentrations [25] and drought [38], among other stressors. It has been suggested that melatonin’s ability to regulate these stresses may be conserved across species. However, this mechanism is not fully replicated in control plants not subjected to stress.

Interestingly, under our experimental conditions, while *CcNiR* and *CcGS2* transcript levels increased in the root, most of these genes were transcriptionally repressed in the leaves. The exception was *CcNiR*, whose transcript levels increased only in the CP treatment. This decrease in gene expression correlated negatively with the fresh and dry weight of the plants. However, GDH activity was differentially regulated according to the melatonin application method; the CP treatment reduced activity, and this reduction correlated positively with nitrate levels and negatively with ammonium levels, while the opposite occurred in the CP + SD treatment.

The discrepancy between transcript levels and growth phenotype may be due to several factors. One of these is that, in the case of *GS*, *GOGAT*, and *GDH*, several sequences encoding these genes were found in the habanero pepper genome, and we evaluated only one member of each, which was selected in the semi-quantitative RT-PCR analyses. For future studies, the transcript levels of the remaining genes should be evaluated.

Another factor could be that melatonin regulates nitrogen metabolism in habanero peppers at another level of regulation, such as post-translational regulation. This appears to be the case for GDH. Nitrogen metabolism enzymes are regulated at multiple levels, including transcript abundance, protein stability, enzyme activation, redox state, and metabolite availability [57]. Since melatonin acts as both an antioxidant and signaling molecule, it may regulate metabolic functionality independently of transcriptional responses.

The behavior of *CcGDH1* was particularly remarkable. In most previously published studies, melatonin simultaneously increases both GDH transcript levels and enzymatic activity, as reported in soybean plants [51]. In contrast, our results revealed increased GDH enzymatic activity despite reduced *CcGDH1* transcript levels, especially under CP + SD treatment. This discrepancy strongly suggests the existence of alternative regulatory mechanisms controlling GDH functionality.

The role of GDH in plant nitrogen metabolism remains controversial. Traditionally, GDH has been considered primarily a catabolic enzyme involved in glutamate deamination [58]. However, increasing evidence indicates that GDH may also participate in ammonium assimilation, nitrogen remobilization, and metabolic adaptation under altered physiological conditions [59]. Several studies have proposed that GDH becomes particularly important when the GS/GOGAT pathway is limited or insufficient.

Melatonin may regulate the growth of habanero peppers through multilevel regulation, for example, by optimizing carbon metabolism. Melatonin increased total carbohydrates in habanero peppers subjected to both application methods. This could indicate improvements in the plant’s photosynthetic capacity, which should be evaluated in further studies. Melatonin has been reported to induce the expression of nitrogen metabolism genes in a pathway independent of those involved in carbon metabolism [25].

Overall, the data suggest that the form of melatonin application can lead to fine differential regulation of nitrogen metabolism in habanero chili leaves and that the melatonin-induced balance of C and N metabolites in chili plants can lead to changes in plant responses to internal growth signals as well as other external environmental signals.

## 4. Materials and Methods

### 4.1. Plant Material, Growth Conditions, and Melatonin Treatments

Seeds of *C. chinense* Jacq. cv. Magnum-Hot Habanero Orange (Genesis Seeds Ltd., Ashalim, M.P. Ramat Negev 8551200, Israel) were used in this study. Seeds were surface-sterilized following the protocol described by [60]. Seeds were then immersed for 48 h at 4 °C in the darkness in either distilled water or melatonin solutions at different concentrations (0.1, 1, 5, 25, 50 and 100 µM) (CP). After treatment, seeds were transferred to Petri dishes (100 seeds per treatment, 25 seeds per dish) containing a thin cotton layer and a sterile filter paper disk moistened with 10 mL of sterile water. The dishes were incubated in darkness at 25 ± 2 °C until germination.

After germination (7 days), germinated seeds were transplanted into 12-well trays (12 plants per tray) filled with peat moss substrate moistened with sterile distilled water. Two trays were used for each treatment.

When the first pair of true leaves had emerged, seedlings received a first fertilization directly in the substrate with Hoagland’s nutrient solution diluted to 1/5 of ionic strength. The solution contained 12.5 µM H_3_BO_3_, 1 µM MnSO_4_·H_2_O, 1 µM ZnSO_4_·7H_2_O, 0.5 µM CuSO_4_·5H_2_O, 0.1 µM H_2_MoO_4_, 0.1 µM NiCl_2_·6H_2_O, 10 µM Fe-EDDHA, 1.20 mM KNO_3_, 0.80 mM Ca(NO_3_)_2_·4H_2_O, 0.40 mM MgSO_4_·7H_2_O and 0.20 mM NH_4_H_2_PO_4_, pH adjusted to 5.8. After one week, the two trays were divided into two groups: (i) seedlings subjected only to melatonin CP, and (ii) seedlings subjected to melatonin chemopriming plus supplementation of the substrate (SD) with the same melatonin concentration used during priming (CP + SD). In this second group, melatonin treatment was applied by adding 15 mL in the substrate per seedling of melatonin solutions at the same concentration used in seed priming, while plants that only underwent melatonin priming were watered with the same volumes of distilled water. Two weeks later, a second application of melatonin was carried out by adding 10 mL per well of both melatonin solution and Hoagland’s nutrient solution (1/5 of ionic strength), while the melatonin-primed-only plants were similarly watered with the equivalent volume of same solution without melatonin. Plants were harvested one week after this treatment. The seedlings were photographed, and the fresh and dry weights of the aerial and root parts were recorded. Tissue of leaves, stems, and roots were collected and stored at −80 °C until subsequent analyses.

### 4.2. Nitrate, Ammonium, Total Amino Acids, Total Protein Contents, Total Carbohydrates, and Proline

One gram of roots, stems, and leaves was used for metabolite extraction, following the procedure described by [44]. Nitrate concentration was determined following the method of [61], using KNO_3_ (Sigma-Aldrich, St. Louis, MO, USA) as a standard. Total amino acids were determined following the methodology of [62]. Total protein content was quantified according to the Bradford assay [63], using the manufacturer’s protocol (Bio-Rad Laboratories, Hercules, CA, USA, Cat. No. 5,000,006). Three biological extractions were performed, and measurements were made in triplicate.

Ammonium concentration was determined colorimetrically using the Nessler reaction according to [64], with modifications. For the colorimetric reaction, plant extract was mixed with Milli-Q water to a final volume of 1 mL. Subsequently, 100 µL of freshly diluted Nessler reagent (1:10, *v*/*v*) was added. Samples were mixed and incubated in the dark at room temperature for 30 min. Absorbance was measured at 385 nm. Ammonium concentration was calculated using a standard curve prepared with ammonium chloride and expressed as mg/g of fresh weight.

Total carbohydrates were quantified using the phenol–sulfuric acid method described by [65]. For the colorimetric reaction, an aliquot of extract was mixed with distilled water to a final volume of 500 µL. Subsequently, 500 µL of 5% (*w*/*v*) phenol and 2.5 mL of concentrated sulfuric acid were added. The reaction mixture was incubated at 30 °C for 20 min to allow color development. Absorbance was measured at 490 nm. Total carbohydrate concentration was determined from a glucose standard curve. The total carbohydrate content of the sample was calculated and expressed as mg/g of fresh weight.

To determine the proline content, the methodology described by [66,67] was used with modifications. The plant material (fresh weight) of the roots and leaves (0.1 g) was macerated and homogenized with 1 mL of 3% (*w*/*v*) sulfosalicylic acid, after which the extract was collected and centrifuged at 14,600× *g* (13,000 rpm) for 10 min. In a new tube, 200 μL of glacial acetic acid, 200 μL of acidic ninhydrin, and 200 μL of plant extract (reaction mixture) were added, and the samples were incubated at 96–100 °C for 60 min. Incubation tubes were then placed on ice to stop the reaction. The extraction of the sample was carried out by adding 1 mL of toluene to the reaction mixture, which was vigorously stirred for 20 s until the organic and aqueous phases were separated. The organic phase containing the chromophore was collected in a quartz cuvette, and absorbance was measured at 520 nm, using toluene as a blank. The Pro concentration was determined from a standard curve and calculated based on the fresh weight (usually expressed as micrograms per gram or micromoles per gram of fresh weight).

### 4.3. Multivariate Analysis of Nitrogen-Related Metabolites

Nitrate, total amino acid, total protein, and ammonium content data obtained from leaves, stems, and roots were analyzed using principal component analysis (PCA) to identify multivariate patterns associated with the treatments. Prior to analysis, the data were scaled to avoid bias caused by differences in variable magnitudes. PCA was performed in RStudio using R software (version 4.4.3, R Foundation for Statistical Computing), and the first two principal components (PC1 and PC2) were selected for interpretation, as they explained the largest proportion of the total variation. PCA results were visualized using scatter plots with 95% confidence ellipses, where points represent individual samples, coded by treatment and melatonin concentration.

### 4.4. In Silico Identification of Nitrogen Metabolism-Related Genes and Primer Design

Genes encoding key enzymes involved in nitrogen metabolism, including *NR*, *NiR*, *GS*, *GOGAT* and *GDH*, were identified in silico using reference sequences retrieved from public databases corresponding to *A. thaliana* (Table S1). These reference sequences were employed as queries in BLASTp searches against the *C. chinense* proteome (assembly ASM227289v2), obtained from the National Center for Biotechnology Information (NCBI, https://www.ncbi.nlm.nih.gov/datasets/genome/) (accessed on 5 June 2025) through the NCBI Datasets Genome platform. BLASTp analyses were performed using command-line tools with default parameters.

Candidate protein sequence were curated to remove redundant entries and subsequently examined for the presence of conserved functional domains using InterProScan (https://www.ebi.ac.uk/interpro/search/sequence/, accessed on 8 August 2025), to validate their functional annotation. Only sequence displaying the characteristic domain architecture described for each enzyme family were retained for subsequent analyses.

The corresponding coding DNA sequences (CDSs) of the selected proteins were retrieved from the *C. chinense* using R software version 4.6.0 (R Foundation for Statistical Computing, Vienna, Austria).

Gene-specific primers were designed based on these CDSs using Primer-BLAST (NCBI, https://www.ncbi.nlm.nih.gov/tools/primer-blast/index.cgi, accessed on 14 August 2025), applying default parameters, and ensuring primer specificity against the *C. chinense* genome.

Additionally, protein sequences from *A. thaliana* and *C. chinense* were compiled to perform a comparative sequence analysis. This analysis included the determination of protein length and molecular weight (kDa) using ExPASy ProtParam (https://web.expasy.org/protparam/, accessed on 15 March 2026). Predicted subcellular localization was assessed using two platform, WoLF PSORT (https://wolfpsort.hgc.jp/, accessed on 15 March 2026) and Plant-mPLoc (http://www.csbio.sjtu.edu.cn/bioinf/plant-multi/, accessed on 15 March 2026). The presence of signal peptide was evaluated using TargetP 2.0 (https://services.healthtech.dtu.dk/services/TargetP-2.0/, accessed on 15 March 2026). The percentage of amino acid identity relative to *A. thaliana* sequences was determined based on previously obtained BLASTP results.

To support gene gamily classification and nomenclature assignment, phylogenetic analyses were conducted for the GS, GOGAT and GDH protein families using amino acid sequences from several plant species, including *C. chinense*, *A. thaliana*, *S. lycopersicum*, *S. tuberosum* and *N. tabacum*. Full-length amino acid sequences corresponding to each family were aligned independently using the MUSCLE algorithm implemented in MEGA software version 12.0.11. with default parameters.

Phylogenetic trees for GS, GOGAT, and GDH proteins were constructed separately using the Neighbor-Joining method based on amino acid sequence alignments. The p-distance model was applied, and gaps were treated using pairwise deletion. The robustness of the inferred phylogenies was assessed by bootstrap analysis with 1000 replicates. Phylogenetic reconstruction was carried out in MEGA software version 12.0.11., and the final tree visualization and graphical customization were performed in R software version 4.6.0 (R Foundation for Statistical Computing, Vienna, Austria).

### 4.5. Total RNA Extraction

Total RNA was extracted from 0.1 g of root and leave tissue using the Plant/Fungi Total RNA Purification kit (Norgen Biotek Corp., Thorold, ON, Canada) following the manufacturer’s instructions. The RNA samples were subsequently treated with Turbo DNAse (Invitrogen, Carlsbad, CA, USA) to remove genomic DNA contamination.

### 4.6. First Strand of cDNA Synthesized

First-strand cDNA was synthesized from (1 µg) total RNA by reverse transcription using the ImProm-II Reverse Transcriptase (Promega, Madison, WI, USA) according to the manufacturer’s instructions.

### 4.7. RT-qPCR Analysis

RT-qPCR analyses were carried out using Maxima SYBR Green/ROX qPCR Master Mix (Thermo Fisher Scientific, Waltham, MA, USA) and gene-specific primers designed for nitrogen assimilation-related genes, including *CcNR*, *CcNiR*, *CcGS*, *CcGOGAT* and *CcGDH* (Table S2). Amplification reactions were performed using a StepOnePlusTM Real-Time PCR System (Thermo Fisher Scientific, Waltham, MA, USA) under the following cycling conditions: initial denaturation at 95 °C for 10 min, followed by 40 cycles of 95 °C for 15 s, 57 °C for 30 s, and 72 °C for 30 s. A melting-curve analysis was conducted at the end of each run to confirm amplification specificity.

Gene expression levels were normalized using tubulin as an internal control, and relative transcript abundance was calculated using the 2^−∆∆Ct^ method [68]. Three independent biological replicates, each with three technical replicates, were analyzed for each tissue and treatment.

### 4.8. Heatmap and Principal Component Analysis of Nitrogen Assimilation Gene Expression

Relative expression data of *CcNR*, *CcNiR*, CcGS1.4, *CcGS2*, *CcFd-GOGAT*, and *CcGDH1* were analyzed by heatmap and principal component analysis (PCA) using R software version 4.4.3 (R Foundation for Statistical Computing, Vienna, Austria). Expression values were log2-transformed [log_2_(x + 1 × 10^−6^)] and standardized prior to analysis.

Heatmaps were generated using the pheatmap package version 1.0.13 with hierarchical clustering based on Ward’s method (“ward.D2”). PCA was performed using the FactoMineR version 2.14 and factoextra version 2.0.0 packages with unit variance scaling enabled. The first two principal components (PC1 and PC2) were used to evaluate sample distribution and gene contribution patterns among treatments and organs.

### 4.9. *GDH* Activity Assay

GDH activity was determined spectrophotometrically at 340 nm by monitoring NADH consumption, according to the methodology described by [69]. The final reaction mixture (1.58 mL) consisted of 1.23 mL of 0.1 M Tris-HCl buffer (pH 8.4), 0.1 mL of 1.5 M ammonium sulfate, 0.1 mL of 0.2 M α-ketoglutarate, and 0.05 mL of 5 mM NADH. The reaction was initiated by adding 0.1 mL of crude enzymatic extract. A reaction mixture without α-ketoglutarate was used as the blank. Enzymatic activity was expressed as µmol NADH consumed min^−1^ mg^−1^ protein. The obtained values represent the mean of triplicate determinations for each evaluated treatment.

### 4.10. Pearson Correlation and Hierarchical Clustering Analysis of Nitrogen Metabolism-Related Variables

Pearson correlation analysis was performed to evaluate the relationships among physiological, biochemical, and molecular variables associated with nitrogen metabolism under the evaluated treatments. Correlation coefficients were calculated in R version 4.4.3 (R Foundation for Statistical Computing, Vienna, Austria) using Pearson’s method. Statistical significance was determined using bilateral probability tests, and correlations were considered significant at *p* ≤ 0.05. The correlation matrix was visualized as a heatmap using the heatmap package version 1.0.13 in R version 4.4.3 (R Foundation for Statistical Computing, Vienna, Austria). Positive and negative correlations were represented using a divergent color scale ranging from blue (negative correlation) to red (positive correlation), with white indicating values close to zero. Hierarchical clustering analysis was applied to both rows and columns using Euclidean distance and complete linkage clustering to identify variables with similar correlation patterns.

### 4.11. Statistical Analysis and Graphical Visualization

All data were subjected to one-way analysis of variance (ANOVA), and differences among means were determined using Tukey’s test (*p* ≤ 0.05) with the SigmaPlot statistical package version 12.0 (Systat Software, Inc., San Jose, CA, USA). Graphical visualization and figure assembly were performed in R software version 4.6.0 (R Foundation for Statistical Computing, Vienna, Austria) [70] using the different packages according to the analysis performed.

## 5. Conclusions

Our study demonstrated that applying 25 and 50 µM melatonin via seed chemopriming stimulated the growth of habanero pepper plants under non-stressful conditions. This effect was maintained when, in addition to priming, the plants received a booster application. Leaves were the organs most sensitive to changes in nitrogen metabolites in response to melatonin treatments, with different application methods having contrasting effects on nitrate and ammonium content. Melatonin also increased proline and carbohydrate levels, and these values correlated with plant weight. At the transcriptional level, melatonin decreased most transcripts of nitrogen metabolism enzymes, while increasing GDH activity in the CP + SD treatment. These results provide new insights into the regulation of nitrogen metabolism according to the application method and dosage, establishing an application strategy to optimize nitrogen metabolism and growth in habanero peppers. Our study underscores the need to investigate the regulatory mechanism of melatonin on N and carbon metabolism and explore whether this impact helps to counteract the effect of environmental stress to optimize its application in agriculture.

## Figures and Tables

**Table 1:** Nomenclature, accession number, and structural characteristics of the nitrogen metabolism proteins in *C. chinense*.

ID Protein	Protein Name	Amino Acids	MW (kDa)	Subcellular Localization	Signal Peptide	*Arabidopsis* Orthologs	ID Protein *Arabidopsis*	Percent Identity
PHU19668.1	CcNR	908	102.08	Cp/Px	NOT	AtNIA2	AT1G37130	75.38
PHU04679.1	CcNiR	585	65.58	Cp/Mit	cTP	AtNiR1	AT2G15620	78.44
PHT99661.1	CcGS1.4	326	35.76	Cyt/Mit	NOT	AtGS1.4	AT5G16570	88.96
PHU30546.1	CcGS2	432	47.61	Cp/Mit	cTP	AtGS2	AT5G35630	85.88
PHU16810.1	CcGS1.2	356	39.24	Cyt	NOT	AtGS1.2	AT1G66200	85.75
PHU23247.1	CcNADH-GOGAT	1583	172.63	Cp	cTP	AtNADH-GOGAT1	AT5G53460	84.67
PHU22565.1	CcFd-GOGAT	413	44.36	Cyt/Cp	NOT	AtFd-GOGAT	AT5G04140	78.56
PHU30311.1	CcGDH	634	70.56	Nu/Mit	NOT	AtGDH	AT1G51720	78.49
PHU06131.1	CcGDH1	441	48.16	Cyt/Mit	NOT	AtGDH1	AT5G18170	83.45
PHU18090.1	CcGDH2	411	48.16	Mit	NOT	AtGDH2	AT5G07440	86.62

Cp: Chloroplast; Px: Peroxisome; Mit: Mitochondrion; Nu: Nucleus; Cyt: Cytoplasm and cTP: Chloroplast transfer peptide.

## Data Availability

The data are included in the manuscript.

## Supplementary Materials

The following supporting information can be downloaded at: https://www.mdpi.com/article/10.3390/plants15111713/s1, Figure S1: Total nitrogen metabolites in leaves (A), stems (B), and roots (C) of *C. chinense* seedlings subjected to different melatonin treatments. Bars represent the sum of nitrate (NO_3_^−^), ammonium (NH_4_^+^), amino acids (AA), and protein (PROT) contents expressed as total nitrogen metabolites (mg g-1 FW). Values represent mean ± SD; Figure S2: Correlation heatmap showing the relationships among physiological, metabolites, and gene expression associated with nitrogen metabolism. Positive correlations are shown in red, negative correlations in blue. Color intensity indicates the strength of the correlation, based on the Pearson correlation coefficient (r); Table S1: Nitrogen metabolism-related proteins from *Arabidopsis thaliana* and their accession number; Table S2: Primer sequence for nitrogen metabolism genes in *Capsicum chinense*.
