# Supplementary material for: Differential Effects of Melatonin on Nitrogen Metabolism and Growth in Capsicum chinense Jacq
Source: Plants (Basel). 2026 Jun 1;15(11):1713. doi: 10.3390/plants15111713 (PMC13259437; doi:10.3390/plants15111713)
Supplement: Supplementary file 1 [file plants-15-01713-s001.zip › Legends Figure S.pdf]

Figure S1. Total nitrogen metabolites in leaves (A), stems (B), and roots (C) of *C. chinense* seedlings subjected to different melatonin treatments. Bars represent the sum of nitrate (NO_3^-), amoonium (NH_4^+), amino acids (AA), and protein (PROT) contents expressed as total nitrogen metabolites (mg g^{-1} FW). Values represent mean ± SD.

Figure S2. Correlation heatmap showing the relationships among physiological, metabolites, and gene expression associated with nitrogen metabolism. Positive correlations are shown in red, negative correlations in blue. Color intensity indicates the strength of the correlation, based on the Pearson correlation coefficient (r).
