# Supplementary figures and images for: Differential Effects of Melatonin on Nitrogen Metabolism and Growth in Capsicum chinense Jacq
Source: Plants (Basel). 2026 Jun 1;15(11):1713. doi: 10.3390/plants15111713 (PMC13259437; doi:10.3390/plants15111713)

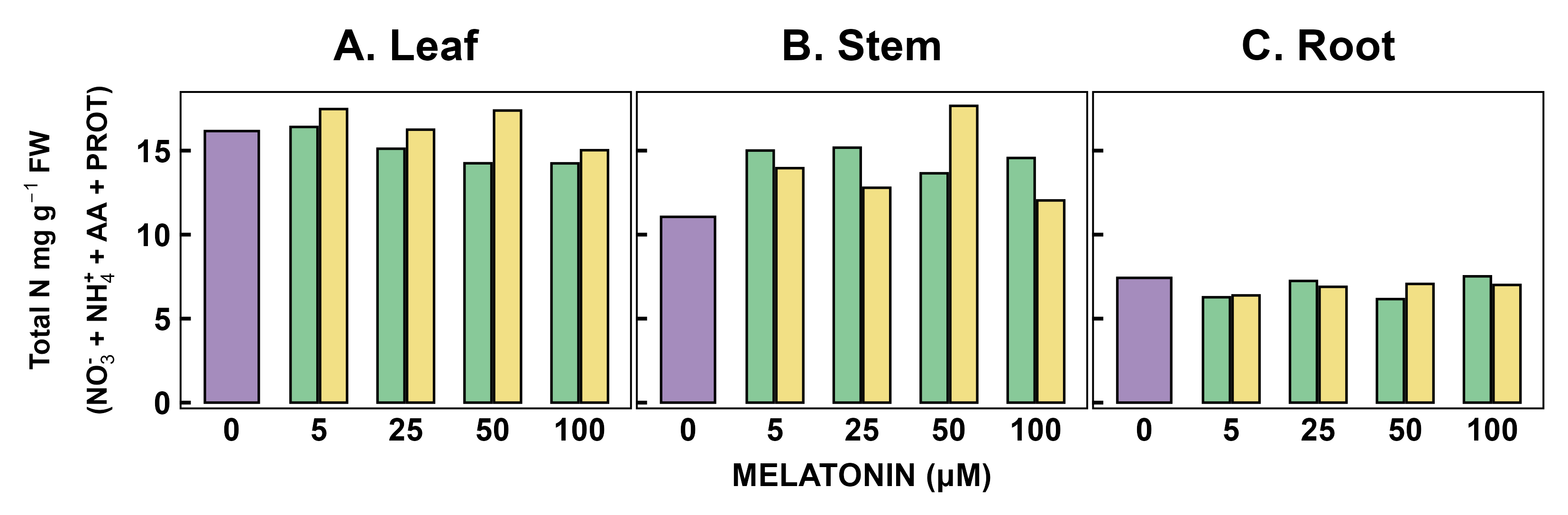

Supplement: Supplementary file 1 [file plants-15-01713-s001.zip › Figure S1.tiff]

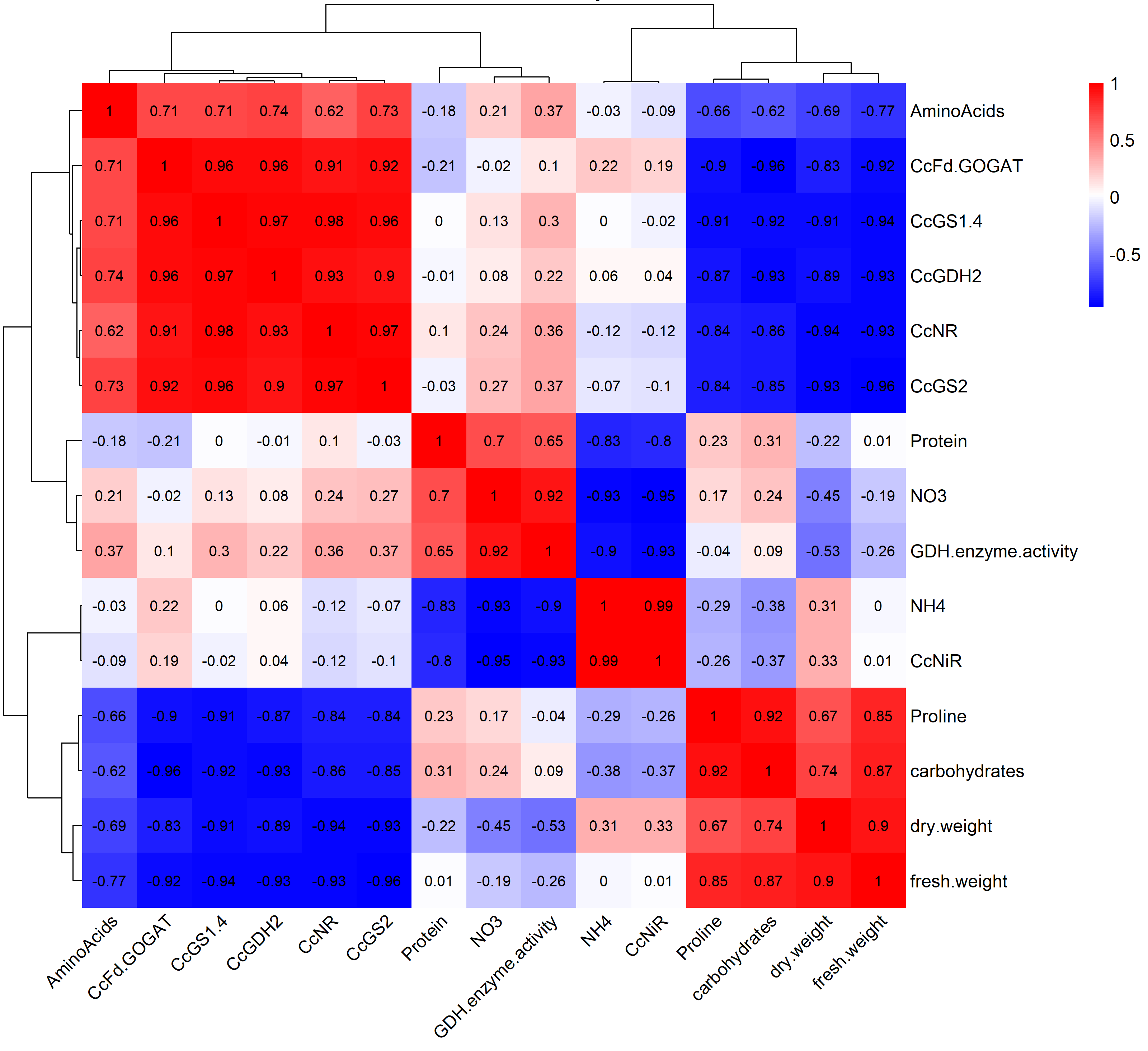

Supplement: Supplementary file 1 [file plants-15-01713-s001.zip › Figure S2.png]
